# Evaluation of the Haemodynamic Behaviour of Stenosed Aortic Heart Valves Using Fluid Structure Interaction Modelling

**DOI:** 10.1002/cnm.70156

**Published:** 2026-03-06

**Authors:** Lindi Grobler Kock, Ryno Laubscher, Johan van der Merwe, Martin P. Venter, Anton F. Doubell, Philip G. Herbst

**Affiliations:** ^1^ Institute of Biomedical Engineering, Department of Mechanical and Mechatronic Engineering Stellenbosch University Stellenbosch South Africa; ^2^ Materials Optimisation and Design, Department of Mechanical and Mechatronic Engineering Stellenbosch University Stellenbosch South Africa; ^3^ Division of Cardiology, Faculty of Medicine and Health Sciences Stellenbosch University Cape Town South Africa

**Keywords:** aortic valve pressure gradients, calcific aortic stenosis, fluid structure interaction, rheumatic aortic stenosis

## Abstract

Aortic stenosis (AS) is a valvular heart disease characterised by the narrowing of the valve opening area. Calcific aortic stenosis (CAS) and rheumatic aortic stenosis (RAS) have distinctly different valve morphologies. The haemodynamic environment of generic calcific and rheumatic aortic valves (AV) of various severities is analysed through the use of 3D FSI modelling techniques. For moderate (AVA = 1–15 cm^2^), severe (AVA < 1 cm^2^) and very severe (AVA ≪ 1 cm^2^) cases of calcific and rheumatic AS, larger TPGs with higher velocity magnitudes are estimated in the rheumatic cases compared to the calcific cases. The additional work required by the left ventricle to overcome the TPG caused by the moderate, severe and very severe rheumatic valve lesions are 5.6%, 42.0% and 58.3% higher compared to the calcific valves of the same severity. The clinical approximation of the TPG is determined according to the simplified Bernoulli approximation and compared to the ground‐truth TPG from the FSI results. The insensitivity of the clinical TPG approximation to the type and severity of stenosis is evident. Overall, the clinical approximation of the TPG either over‐ or underpredicts the TPG depending on the type and severity of the lesion, with smaller errors in the rheumatic cases compared to the calcific cases.

## Graphical abstract

The haemodynamic environment of generic calcific and rheumatic aortic valves of various severities is analysed through the use of 3D FSI modelling techniques. For the same severity and similar flow rates, the rheumatic valves offer higher resistance to the flow of blood compared to the calcific valves.

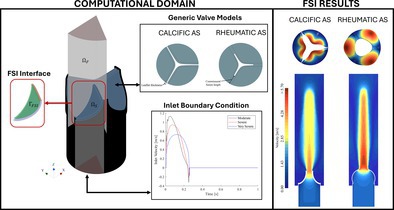

## 1 Introduction

Aortic stenosis (AS) or the narrowing of the aortic valve (AV) orifice is the most common valvular lesion encountered in cardiology. Assessing the valve area and haemodynamic impact of the valve lesion is central to the management of this condition [1, 2]. Calcific aortic stenosis (CAS), commonly seen in older patients, is the most prevalent cause of AS seen in high income countries [3]. In low‐ and middle income countries, including most countries in Sub‐Saharan Africa, rheumatic aortic stenosis (RAS), a late sequelae of rheumatic fever and rheumatic heart disease (RHD), is also a frequent case of AS. RHD is considered the most frequent cause of primary valvular heart disease (VHD), where up to 40 million people were affected in 2019 with an incidence of over 2 million per year [4]. Bicuspid aortic valve (BAV) is the most common congenital heart defect causing AS [5] however, the majority of AS cases seen in South Africa are caused by CAS or RAS.

The severity of AS is determined by the effect that the pathology has on the subsequent haemodynamic environment of the AV and is diagnosed by evaluating the transvalvular pressure gradient (TPG), the velocity profile of the blood flowing through the valve, the geometric and haemodynamic valve opening areas, and the left ventricular functional parameters in terms of ejection fraction, stroke volume (SV) and evidence of compensatory hypertrophy or dysfunction [1, 2]. These geometrical and flow parameters are often determined through a combination of both invasive and non‐invasive examination techniques. However, invasive cardiac catheterisation is only recommended in symptomatic cases with inconclusive non‐invasive findings [1, 2, 5]. The classification ranges for these parameters are outlined in the guidelines published by the American College of Cardiology and the American Heart Association (ACC/AHA), and the European Society of Cardiology and European Association for Cardio‐Thoracic Surgery (ESC/EACTS) [1, 2].

When considering the two main causes of AS in South Africa, it is important to note that the pathology causing a reduction in AV area (AVA) results in differing valve morphologies, including the shape of the valve orifice, thereby impacting the TPG and the velocity profile of blood flowing through the valve. CAS is caused by progressive fibrosis and calcification of the leaflets, resulting in the semilunar leaflets becoming immobile and fixed in systole. The reduction in AVA due to CAS is characterised by the residual slit‐like opening of the commisure between the leaflets. RHD leads to commissural fusion of the leaflets, and the resulting reduced AVA found in RAS is characterised by a central triangular shaped orifice [6, 7].

During systole, blood propelled out of the left ventricle through the systemic loop leads to a pressure differential across the aortic heart valve that causes the valve to open and close rhythmically. In the case of AS, the narrowing of the valve opening area leads to an increased resistance to the flow of blood which influences the mechanical work demand of the LV and results in an elevated TPG. As the relationship between the cardiac output (CO) and the pressure gradient across a stenosed valve is non‐linear, it is important to determine the true TPG when diagnosing AS [8, 9]. In a clinical setting, the anatomical and flow parameters of the defective valve are often analysed through transthoracic echocardiography (TTE) with 2D imaging and Doppler interrogation with the Doppler beam orientation parallel to the AV blood jet. Through Doppler spectral analyses, the velocity profile is generated, and the TPGs are estimated according to a simplified Bernoulli correlation [1, 2]. This correlation is empirically manipulated and derived from the Bernoulli equation for steady, incompressible and inviscid flow and relates the peak and mean jet velocity to the peak and mean TPG [10]. The uncertainty of the TPG estimation through the recommended Bernoulli correlation is well documented in recent studies [8, 9, 11, 12, 13, 14, 15, 16, 17, 18] where efforts have been made to improve the accuracy through numerical and computational approaches. It is evident from the literature that the accuracy of the clinical TPG estimation in RHD and RAS cases is limited.

Studies where the TPG is estimated using steady‐state computational fluid dynamics (CFD) approaches often consider the valve at peak systole conditions where either the peak or mean velocity conditions are used [8, 17, 18]. This results in peak TPG simulated measurements instead of the mean TPG diagnostic parameter or a mean TPG that is estimated at a peak AVA. As the AVA dynamically changes during systole, and there exists a non‐linear relationship between the TPG and the velocity profile, it is possible that the mean TPG cannot be accurately estimated at peak systole (and peak AVA) conditions. From a previous study by the authors, steady‐state CFD simulations for various magnitudes of CO indicated that there is a discrepancy between the haemodynamic environment of CAS and RAS of the same severity when considering the simulated TPG, the magnitude and location of the peak jet velocity (and therefore the location of the effective orifice area—EOA), and the corresponding clinical estimation of the TPG [8].

The simplifying assumptions associated with steady‐state CFD analyses warranted further investigation using more complex modelling techniques. To overcome the limitation of steady‐state CFD analyses of a compliant AV, computational fluid–structure interaction (FSI) approaches are used. Through the use of FSI models, the dynamic behaviour of the deforming leaflets and the momentum and dampening of the blood flow are captured, allowing for a more accurate representation of the haemodynamic environment of the diseased AVs. In cardiovascular biomechanics, FSI has gained popularity due to the inclusion of both the structural and fluid domains when analysing the haemodynamics of the aortic heart valve [19, 20]. More specifically, FSI models of the aortic heart valve include that of healthy native, synthetic or mechanical, and pathological valves mostly pertaining to calcific and bicuspid VHD [19, 20, 21, 22, 23, 24, 25, 26, 27, 28, 29]. To the best of the authors' knowledge, there is no substantial literature regarding FSI modelling of RHD and RAS where the haemodynamic environments of the two AS pathologies are studied and compared to CAS simulated results.

The current work analyses the TPG and velocity profiles of the blood flow through generic calcific and rheumatic stenosed AVs by developing FSI models. For the same severity of AS, the effect of the different disease morphology on the AV's haemodynamic environment is evaluated. This includes the peak and mean TPGs, the peak and mean jet velocities, the location of the EOAs and the lost work due to the pressure drop across the valves. Furthermore, the velocity profile at the EOA is used to estimate the corresponding peak and mean TPG according to the clinical Bernoulli equation and compare it to the corresponding simulated TPGs from the FSI models. The results from this numerical study provide a better understanding of the haemodynamic environment of the AV in varying degrees of both calcific and rheumatic stenosed conditions and enable further discussion on the accuracy of the clinical TPG estimation in both disease types.

## 2 Materials and Methods

### 2.1 Description of Computational Domain

**FIGURE 1:**
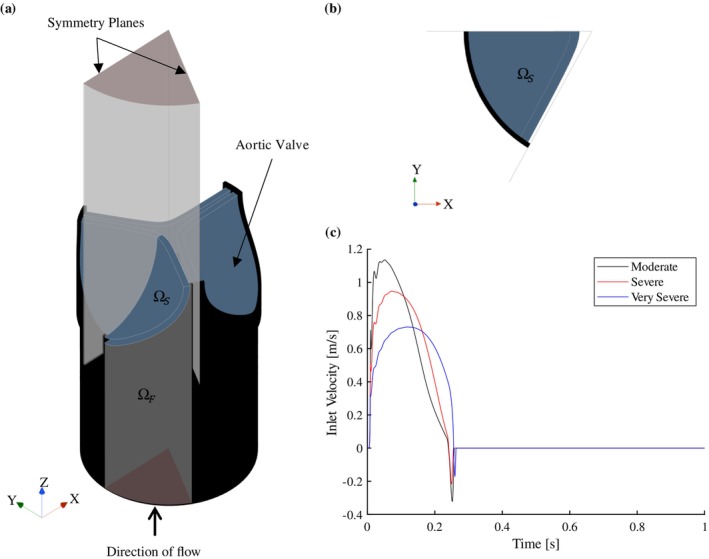
Computational domain: (a) fluid and solid domain sectioning according to the symmetry conditions and the symmetry planes; (b) top‐view of the solid domain and symmetry planes and (c) moderate, severe and very severe velocity inlet boundary condition.

The computational domain assumes that the left ventricular outflow tract (LVOT), AV and aortic root are symmetrical about the axial axis. Therefore, of the entire three‐leaflet AV, the computational domain only considers one‐sixth of the valve, i.e., half of one leaflet as illustrated in Figure 1a,b, where symmetry planes are used to create the full domain. The fluid domain $\Omega_{f}$ includes the aortic root (downstream from the leaflet) modelled as rigid walls, a moving wall at the fluid–solid interface, a velocity profile at the inlet boundary condition (BC) shown in Figure 1c, and a constant pressure outlet BC [27, 30, 31]. In the current work, the compliance of the aortic root is disregarded in order to investigate the valve's haemodynamic effect in isolation. The fluid domain shown in Figure 1a does not represent the entire downstream and upstream regions. For a heartrate of $60\;\text{beat}\cdot \min^{-1}$, the velocity profile over time for a single heartbeat of the cardiac cycle is shown in Figure 1c. These transient velocity profiles are generated for moderate, severe and very severe cases of AS using the lumped parameter model (LPM) developed by Laubscher et al. [32]. The solid domain $\Omega_{s}$ consists of a solid leaflet fixed within the LVOT.

### 2.2 Case Studies

**FIGURE 2:**
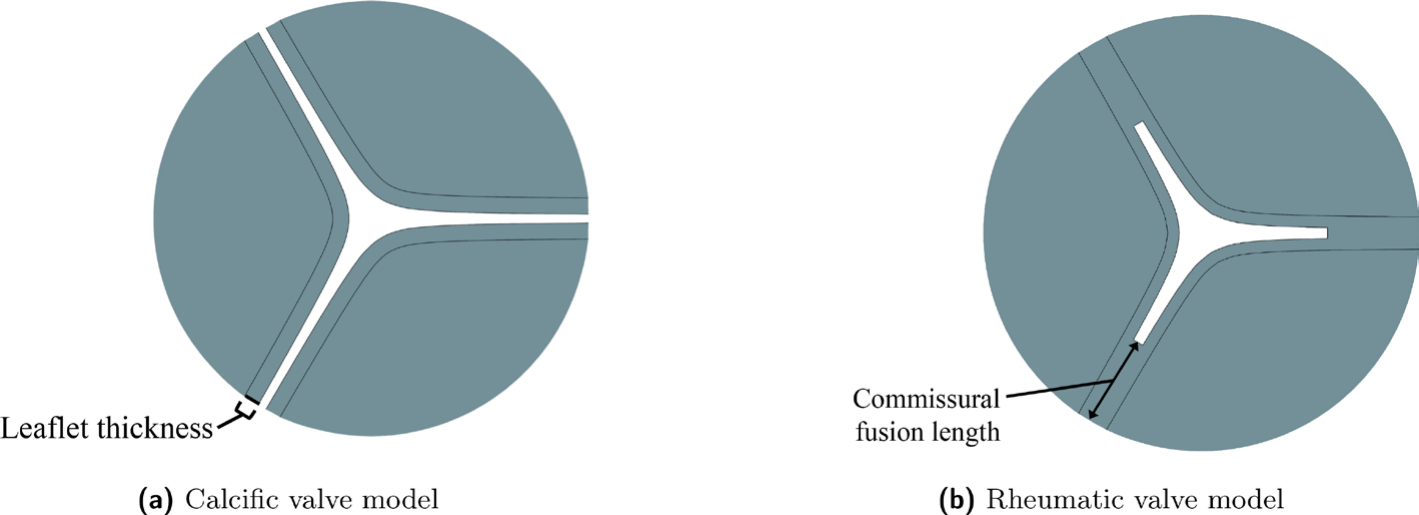
Leaflet thickening and commissural fusion evident in the moderate: (a) calcific and (b) rheumatic AS valve models.

The generic valve model [33, 34] in each case study is based on the characteristics of the respective pathology. Similar generic pathological valve models were introduced in a previous study by the authors of the current work [8]. To represent the calcification of the leaflets in CAS cases, the leaflets are modelled as uniformly thickened structures where the thickness increases with an increase in severity [35]. The commissural fusion evident in RAS cases is mimicked by the fusion of a healthy native valve's leaflet edges [7]. The moderately stenosed valve models are shown in Figure 2 where the thickening of the leaflets in the calcific case is indicated in Figure 2a, and the increased commissural fusion length in the rheumatic case is indicated in Figure 2b. It is important to note that the leaflet thickness in the RAS cases is considered to be that of a native valve (0.65 mm), as the combined effect of calcification and commissural fusion is not considered in the current work. As a result, the characteristics of each pathology is evaluated in isolation, and its effect on the haemodynamic environment of the AV is analysed. The valve models are created during the FSI simulations according to the AVA measured at peak systole (i.e., peak BC and EOA velocity) for each severity which ranges between 1 and 1.5 cm^2^ for moderate AS, and < 1 cm^2^ for severe and very severe AS [1, 2]. The geometric valve opening area ratio (AR) is determined by the ratio of the AVA to the area of the LVOT at the base of the valve. Therefore, the AR at peak systole is governed by the severity of the disease, and is used to determine the thickness of the leaflets in the CAS cases and the fusion lengths in the RAS cases. The geometric parameters, AVAs and ARs at peak systole for each valve model are summarised in Table 1. The resultant moderate, severe and very severe calcific and rheumatic valve models at peak systole conditions are discussed in Section 3. These valve models are included in the FSI simulations as deforming solid structures.

**TABLE 1:** Moderate, severe and very severe CAS and RAS valve model characteristics at peak systolic flow conditions.

Type	Severity	Leaflet thickness (mm)	Commissural fusion length (mm)	AVA (cm^2^)	AR
CAS	Moderate	0.896	—	1.494	0.330
	Severe	1.180	—	0.992	0.219
	Very severe	1.950	—	0.604	0.134
RAS	Moderate		5.05	1.516	0.335
	Severe	0.650	7.75	1.004	0.222
	Very severe		10.65	0.626	0.138

### 2.3 Modelling Theory

An FSI simulation is governed by the principle that the fluid and solid continua deform with the same kinematics and experience the same traction at the fluid–structure interface [36]. At the fluid–structure interface, fluid traction is transferred from the fluid domain to the solid domain resulting in deformation of the solid domain. Deformation of the solid domain in the form of displacement is consecutively transferred to the fluid domain, resulting in a two‐way coupled FSI simulation. The FSI algorithm follows a partitioned approach where the fluid and structural equations are solved separately. The fully coupled fluid and structural solvers require a concurrent solution strategy and are formulated implicitly in time. The time dependent fluid domain ($\Omega_{f}\left(t\right)$) is solved within the fluid solver (F) and the time dependent structural domain ($\Omega_{s}\left(t\right)$) is solved within the structural solver (S). To enable a two‐way coupled FSI model of the blood flowing through and deforming the AV, contact interfaces ($\Gamma_{\mathrm{FSI}}$) between the structural and fluid surfaces at the valve are created [20, 31].

In essence, the flow equations are solved for a given displacement (*u*) of the contact interface $\Gamma_{\mathrm{FSI}}$ as a Dirichlet BC. The structural equations are solved for a given traction (*T*) at the contact interface $\Gamma_{\mathrm{FSI}}$ as a Neumann BC. Therefore, at $\Gamma_{\mathrm{FSI}}$, the respective domains are solved according to Equation (1):

(1) $$\begin{array}{l} \left(a\right)\;T=F\left(u\right)\\ \left(b\right)\;u=S\left(T\right) \end{array}$$

#### 2.3.1 Fluid Flow Conservation Equations

Blood as the working fluid is considered incompressible and assumed to exhibit Newtonian behaviour at the geometric scale modelled in the present work [12, 13, 23, 24, 26, 28, 37]. The finite volume fluid flow solver (F) is based on the incompressible Navier–Stokes equations for Newtonian fluids [36, 38]. The velocity and pressure fields are determined by the conservation of mass and momentum equations shown in Equation (2) for position vector $\vec{x}\in \Omega_{f}\left(t\right)$. Note the momentum equations below are the Reynolds‐averaged Navier–Stokes formulation.

(2) $$\begin{array}{l} \left(a\right)\;\nabla \cdot \bar{U}=0\\ \left(b\right)\;\rho_{f}\left(\frac{\partial \bar{U}}{\partial t}+\nabla \cdot \left(\bar{\mathrm{UU}}\right)\right)=-\nabla \bar{p}+\nabla \cdot \left(\bar{\bar{\tau }}-\rho_{f}\bar{U^{\prime }U^{\prime }}\right)+{\bar{S}}_{f} \end{array}$$

In Equation (2), $\bar{U}=\bar{u}i+\bar{v}j+\bar{w}k$ is the velocity vector in the *x*, *y* and *z* directions respective, $\rho_{f}$ is the fluid density, $\bar{p}$ is the fluid static pressure and ${\bar{S}}_{f}=\rho_{f}\bar{g}+\bar{F}$ is the body force vector per unit fluid volume. The viscous stress tensor $\bar{\bar{\tau }}$ is given in Equation (3) where $\mu_{f}$ is the viscosity of the fluid.

(3) $$\bar{\bar{\tau }}=\mu_{f}\left[\left(\nabla U+\nabla U^{T}\right)-\frac{2}{3}\nabla \cdot \mathrm{UI}\right]$$

The Reynolds stresses arising due to turbulent velocity fluctuations $\left(-\rho_{f}\bar{U^{\prime }U^{\prime }}\right)$ in the momentum Equation (2b) are closed using a turbulence model and the Boussinesq approximation shown in Equation (4) where the turbulent viscosity is denoted $\mu_{t}$ and the turbulent kinetic energy $k=\frac{1}{2}\left(\bar{{u^{\prime }}^{2}}+\bar{{v^{\prime }}^{2}}+\bar{{w^{\prime }}^{2}}\right)$. The specifics of the turbulence model used in the present work will be discussed later.

(4) $$-\rho_{f}\bar{U^{\prime }U^{\prime }}=\mu_{t}\left(\nabla U+\nabla U^{T}\right)-\frac{2}{3}\rho_{f}k\delta_{\mathrm{kk}}$$

#### 2.3.2 Structure Mechanics Conservation Equations

The structural solver (S) uses the finite element method to solve the conservation of mass and momentum equations of the solid continuum and the constitutive equation of the material model [36, 39]. For the position of a material point in the solid continuum in the undeformed configuration X and in the deformed configuration $x\left(X,t\right)$, the deformation of displacement vector is given by Equation (5):

(5) $$u\left(X,t\right)=x\left(X,t\right)-X$$

As a measure of the deformation change between points in the continuum, the deformation gradient tensor $F=\frac{\partial x}{\partial X}$ is defined and its determinant $J=\det\left(F\right)$. The motion of a solid body is governed by Cauchy's equilibrium where the displacement (u) of a solid body for $\vec{x}\in \Omega_{s}\left(t\right)$ is expressed according to the Lagrangian form of the law of conservation of momentum in Equation (6):

(6) $$\rho_{s}\frac{D^{2}\bar{u}}{{\mathrm{Dt}}^{2}}=\nabla \cdot \sigma_{s}+{\bar{S}}_{s}$$

For the second material derivative in Equation (6), $\rho_{s}$ is the density of the solid, $\bar{u}$ is the solid displacement vector in the *x*, *y* and *z* direction, $\sigma_{s}$ is the Cauchy stress tensor and ${\bar{S}}_{s}$ is the body force vector on the solid per unit volume. In the deformed configuration, $\sigma_{s}$ is a measure of the traction acting on the surfaces of the solid body, and the second Piola–Kirchhoff stress tensor (S) refers to the stress in the undeformed configuration. These tensors are related according to Equation (7):

(7) $$S=JF^{-1}\sigma_{s}F^{-T}$$

The stress in a solid is related to the strain according to the constitutive relation of the material model. For a hyperelastic material model, the general strain–stress relationship is given by Equation (8) where S is the second Piola–Kirchhoff stress tensor, $C=F^{T}\cdot F$ is the right Cauchy–Green deformation tensor, and Ψ is the strain energy density function of the material described by the constitutive model.

(8) $$S=2\frac{\partial \Psi }{\partial C}$$

To ensure kinematic equilibrium between the fluid and solid domains with no‐slip conditions at $\Gamma_{\mathrm{FSI}}$, the velocity vector of the fluid has to be equal to the velocity vector of the solid in Equation (9) [20]:

(9) $$\bar{U}=\frac{\partial \bar{u}}{\partial t}$$

Similarly, the traction of the fluid and the solid at $\Gamma_{\mathrm{FSI}}$ is in equilibrium according to Equation (10). Here, the fluid stress tensor is $\sigma_{f}=-pI+\bar{\bar{\tau }}$, and ${\vec{n}}_{f}$ and ${\vec{n}}_{s}$ are the respective unit normal vectors of $\Omega_{f}\left(t\right)$ and $\Omega_{s}\left(t\right)$:

(10) $$\sigma_{f}\cdot {\vec{n}}_{f}=\sigma_{s}\cdot {\vec{n}}_{s}$$

Therefore, for $\vec{x}\in \Gamma_{\mathrm{FSI}}\left(t\right)$, Equations (9) and (10) are valid.

### 2.4 Model Development

**FIGURE 3:**
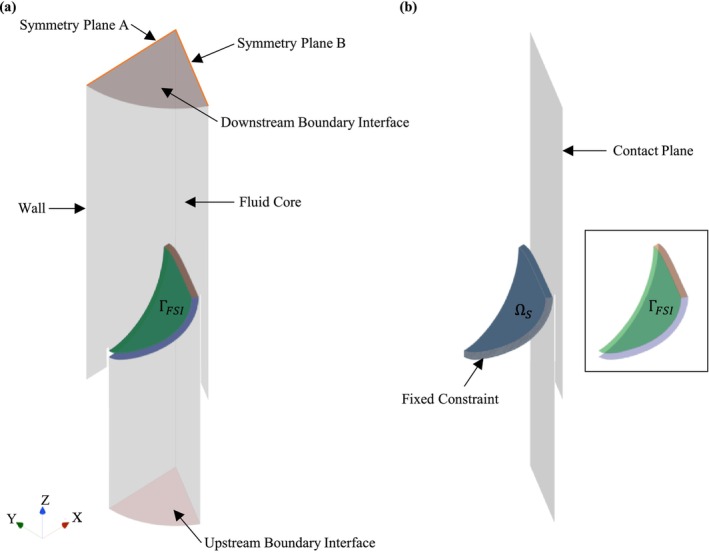
Model development. (a) Fluid ($\Omega_{f}$) and (b) solid ($\Omega_{s}$) domain.

The fluid solver (F) and structural solver (S) are coupled in a partitioned approach in Siemens STAR CCM+ (2402 build 19.02.009, Siemens Digital Industries Software, Plano, TX, USA [36]). The arbitrary Lagrangian–Eulerian (ALE) technique is used to account for the solid displacement of $\Gamma_{\mathrm{FSI}}$ in the fluid domain, where the fluid mesh is deformed and re‐meshed to conform to the solid domain [28, 31, 36]. A first‐order unsteady FSI model with a constant time step solves for a heartrate of $60\;\text{beat}\cdot \min^{-1}$ and cardiac cycle of 1.0 s. The time step size depends on the case and ranges between 0.08 and 0.2 ms as a smaller time step is required to solve the FSI models with larger deformation gradients. A dynamic stabilisation method that adds force correction that is proportional to the displaced fluid per unit area as a pre‐condition to the FSI interface is enabled to improve stability and ensure convergence at each iteration. The structural solver is converged for absolute residuals of force and displacement below 1E−5. The fluid solver is converged for absolute residuals of continuity, momentum and turbulence below 1E−6. The respective domains and their associated boundaries are discussed in Sections 2.4.1 and 2.4.2 and are shown in Figure 3.

#### 2.4.1 Fluid Domain

Blood is modelled as an incompressible Newtonian fluid with a density and dynamic viscosity of $995\;\mathrm{kg}\cdot m^{-3}$ and 0.0035Pa⋅s, respectively [8, 32, 37, 40, 41]. The fluid solver uses a SIMPLE implicit scheme with a realisable two‐layer *k*–*ϵ* turbulence model and blended wall functions. As shown in Figure 3a, the fluid domain has rigid walls with no‐slip conditions, two symmetry plane BC's (A and B), a velocity inlet BC at the LVOT (shown in Figure 1c), a constant pressure outlet BC of 0 Pa, and a no‐slip displacement contact interface at $\Gamma_{\mathrm{FSI}}$. For the purpose of meshing and re‐meshing efficiency, the fluid domain is divided into three regions; namely *Fluid core*, *Downstream* and *Upstream*.

The *Fluid core* region shown in Figure 3a is discretised with polyhedral cells according to a mesh base size and target face size equal to the thickness of the leaflet. The polyhedral mesh is governed by a minimum cell surface size ranging between 10% and 25% relative to the base size (depending on the case) and has a volume growth rate of 1.1. A two‐layer prismatic cell layer with a thickness of 32% relative to the base size is generated at $\Gamma_{\mathrm{FSI}}$ and the fluid walls. To conform the fluid vertices to the displaced solid structure, a new fluid mesh with a minimum face quality of 0.2 is generated in the Fluid Core region when the volume of a cell changes with 25%–40% (depending on the case) between time steps.

The *Downstream* and *Upstream* regions are generated from the respective downstream and upstream surfaces of *Fluid core* region as 200 mm surface extrusions with the same fluid properties. As a result, boundary‐type interfaces indicated in Figure 3a between the respective fluid–fluid regions are generated. The extended regions are discretised with 160 layers of hexahedral cells where the cell sizes conform to the cell size of the neighbouring cells at the boundary interfaces (while the thickness of the cells remains constant). Therefore, the velocity inlet BC is applied at the upstream surface of the *Upstream* region (i.e., the LVOT), and the pressure outlet BC is applied at the downstream surface of the *Downstream* region. Each FSI model has a total fluid mesh size ranging between 135,000 and 220,000 cells, with 90% of the cells with a cell quality > 0.3 and a skewness angle < 40°.

#### 2.4.2 Solid Domain

The valve models described in Section 2.2 are included in the solid domain as shown in Figure 3b. The solid domain is modelled as a hyperelastic, isotropic and homogeneous material with a density of $1000\;\mathrm{kg}\cdot m^{-3}$. A Neo‐Hookean material model in Equation (11) is used with a bulk modulus ($k_{b}$) of 53 MPa and a C_10_ parameter of 0.1666 MPa [8, 25, 26] where $I_{1}^{d}$ is the first invariant of the deviatoric right Cauchy–Green deformation tensor and *J* is the determinant of the deformation gradient tensor from Section 2.3.2.

(11) $$\Psi =C_{10}\left(I_{1}^{d}-3\right)+\frac{k_{b}}{2}{\left(J-1\right)}^{2}$$

Depending on the thickness of the leaflet, the solid domain is discretised with 9500–22,000 cells quadrilateral elements with a minimum cell quality of 0.6 and a maximum skewness angle < 7°. The leaflet has a fixed constraint at the base where it is attached to the LVOT as shown in Figure 3b, and a solid displacement contact interface at the $\Gamma_{\mathrm{FSI}}$ surfaces. To ensure the leaflet deforms within the periodic symmetry boundaries of the computational domain, a displacement constraint in the direction perpendicular to symmetry plane A is applied at the side edge of the leaflet. To ensure the leaflet does not penetrate the imagined opposing leaflet, a frictionless contact plane at symmetry plane B with a gap offset of 0.2 mm to the inner surface of the leaflet is created as shown in Figure 3b. This prescribed clearance between the solid domain and the symmetry plane of the fluid domain is important to prevent the leaflet from deforming the fluid cells to zero volume.

#### 2.4.3 Post Processing of Simulation Results

The simulated FSI results are analysed in MATLAB R2024a [42] to extract important haemodynamic values such as pressure gradients, SV and lost work. The equations and parameters used to extract these and other important values will be discussed below.

The pressure gradient in Pa is calculated at each time step as the difference between the mass flow averaged total pressure at the inlet and at the outlet of the fluid domain. All peak and mean TPG measurements in this text are converted to units of mm⋅Hg by dividing the pressure in Pa by $133.33\;\mathrm{mm}\cdot \mathrm{Hg}\cdot {\mathrm{Pa}}^{-1}$. The velocity profiles in $m\cdot s^{-1}$ at the LVOT (i.e., the inlet BC due to fluid incompressibility) and at the EOA are evaluated. The peak and mean velocity conditions are determined from the velocity profiles as the maximum and the integral with respect to time, respectively. The EOA represents the area within a 4 mm radius from the centre of the domain where the velocity reaches its maximum. This prescribed radius represents the approximate diameter of an echo‐Doppler ultrasound probe aligned parallel to the velocity jet [43].

The peak TPG from the FSI data is denoted ${\mathrm{TPG}}_{\mathrm{FSI}}$ and represents the maximum TPG calculated over the systolic period ($T_{\mathrm{SYS}}$). The mean TPG from the FSI results, denoted $\bar{{\mathrm{TPG}}_{\mathrm{FSI}}}$, is calculated as the time integral of the TPG over the systolic period in Equation (12):

(12) $$\bar{{\mathrm{TPG}}_{\mathrm{FSI}}}=\frac{1}{T_{\mathrm{SYS}}}\int_{T_{\mathrm{SYS}}}\mathrm{TPG}\left(t\right)\mathrm{dt}$$

The peak velocity at the EOA ($V_{\mathrm{EOA}}$) is determined as the maximum velocity in the domain at timestamp $t_{@V_{\mathrm{EOA}}}$ and is considered the time of peak systole. The TPG at peak systole is denoted ${\mathrm{TPG}}_{\mathrm{PS}}$. The mean velocity is calculated as the velocity time integral (${\mathrm{VTI}}_{\mathrm{EOA}}$) of the jet velocity $V_{\mathrm{EOA}}\left(t\right)$ over the systolic period $T_{\mathrm{SYS}}$ (time from valve opening to closing) according to Equation (13):

(13) $${\mathrm{VTI}}_{\mathrm{EOA}}=\frac{1}{T_{\mathrm{SYS}}}\int_{T_{\mathrm{SYS}}}V_{\mathrm{EOA}}\left(t\right)\mathrm{dt}$$

The timestamp associated with ${\mathrm{VTI}}_{\mathrm{EOA}}$ is denoted $t_{@{\mathrm{VTI}}_{\mathrm{EOA}}}$. The clinical TPG approximation is determined according to the simplified Bernoulli equation in Equation (14) where ΔP is the clinical TPG in mm⋅Hg and *V* is the jet velocity in $m\cdot s^{-1}$ measured at the EOA.

(14) $$\Delta P=4\times V^{2}$$

The peak and mean TPGs, according to the clinical estimations, are therefore a function of the peak and mean velocities at the EOA and are denoted ${\mathrm{TPG}}_{\text{Clinical}}$ and $\bar{{\mathrm{TPG}}_{\text{Clinical}}}$, respectively, in Equation (15):

(15) $$\begin{array}{l} \left(a\right)\;{\mathrm{TPG}}_{\text{Clinical}}=4\times V_{\mathrm{EOA}}^{2}\\ \left(b\right)\;\bar{{\mathrm{TPG}}_{\text{Clinical}}}=4\times {\mathrm{VTI}}_{\mathrm{EOA}}^{2} \end{array}$$

The mass flow rate in $\mathrm{kg}\cdot s^{-1}$ in the domain is measured at each time step and is converted to volume flow rate (*Q*) in $\mathrm{mL}\cdot s^{-1}$ and CO in $L\cdot \min^{-1}$. The mean flow rate ($\bar{Q}$) in $\mathrm{mL}\cdot s^{-1}$ over the systolic period is calculated as the time integral of *Q* over $T_{\mathrm{SYS}}$ in Equation (16):

(16) $$\bar{Q}=\frac{1}{T_{\mathrm{SYS}}}\int_{T_{\mathrm{SYS}}}Q\left(t\right)\mathrm{dt}$$

To quantify the impeding effect of the valve on the flow of blood, the lost work due to the pressure drop across the valve is calculated according to Equation (17) as the time integral of power (the product of TPG and *Q*) in *J* over the systolic period.

(17) $$W_{\text{lost}}=\int_{T_{\mathrm{SYS}}}\mathrm{TPG}\left(t\right)\times Q\left(t\right)\mathrm{dt}$$

For a heartrate of $60\;\text{beat}\cdot \min^{-1}$, the blood volume pumped out of the LV at each beat is called the SV measured in $\mathrm{mL}\cdot {\text{beat}}^{-1}$. The SV of a cardiac cycle is calculated from the time integral of *Q* over one heartbeat (*T* = 1.0 s) according to Equation (18):

(18) $$\mathrm{SV}=\int_{T}Q\left(t\right)\mathrm{dt}$$

## 3 Results and Discussion

The FSI models developed for moderate, severe and very severe cases of CAS and RAS using the methods described in Section 2 is presented in this section. A grid convergence study is included in Section 3.1 followed by Section 3.2 that describes the necessary interpretation of the simulated FSI results presented in Sections 3.3 and 3.4. For the same degree of stenosis, the CAS and RAS results are compared in Section 3.5. In this section, subscripts MC, SC and VSC refers to moderate, severe and very severe CAS cases respectively. Similarly, MR, SR and VSR refers to moderate, severe and very severe RAS cases, respectively. The subscript PS refers to peak systole as defined in Section 3.2.

For the results discussed in this section, it is important to reiterate that the combined effect of commissural fusion and leaflet thickening is not considered in the RAS cases. RAS are represented by native leaflets with varying degrees of fused commissures, while CAS are represented by varying degrees of uniformly thickened leaflets.

### 3.1 Mesh Convergence

A grid convergence study is performed on the initial fluid mesh of each FSI model to determine the optimal mesh size where the accuracy of the solution becomes independent of the computational grid [44]. The peak velocity and mean TPG at the EOA are used as the convergence criteria where a grid convergence index (GCI) in each case is calculated for a safety factor of 1.5 and a grid refinement ratio between 1.2 and 1.8. The mesh convergence results are summarised in Table 2. A maximum GCI of 1.85% for the peak velocity and 1.18% for the mean TPG is calculated in the moderate calcific case. As these GCI magnitudes are smaller than the recommended 4%, the fluid meshes used in the FSI simulations are therefore considered converged and the solution sufficiently independent of the computational grids for the current study.

**TABLE 2:** Mesh independence study.

	Total cell count	Peak velocity GCI	Mean TPG GCI
MC	219,715	1.85	1.18
SC	161,259	0.74	0.02
VSC	135,160	0.01	0.29
MR	189,563	0.82	0.18
SR	199,343	0.28	0.15
VSR	196,378	1.17	0.31

### 3.2 Data Interpretation

The following data interpretation methodology applies to all case studies presented below:

1. The systolic period ($T_{\mathrm{SYS}}$) is calculated from the inlet velocity BC as the time when the velocity profile is positive. For the moderate, severe and very severe CAS and RAS cases, the systolic periods are 0.2403, 0.2413 and 0.2554 s, respectively.
2. With the exception of SV, all other data only pertains to the systolic period of the cardiac cycle. For the moderate, severe and very severe CAS and RAS cases, the SV is 71.1, 72.3 and 68.1 mL · beat^−1^, respectively.
3. As the valve opens, the AR rapidly increases and fluctuates before it settles, whereafter it increases and decreases proportionally to the BC velocity profile. The valve is considered *open* after the AR has settled.
4. Peak haemodynamic conditions are determined after the valve is considered open.
5. Mean haemodynamic conditions are calculated over the entire systolic period according to Equations (12), (13) and (16).
6. The EOA velocity in the domain increases as the valve opens and reaches a maximum ($V_{\mathrm{EOA}}$). The time of peak EOA velocity ($t_{@V_{\mathrm{EOA}}}$) describes the phrase *peak systole*.

### 3.3 CAS

**FIGURE 4:**
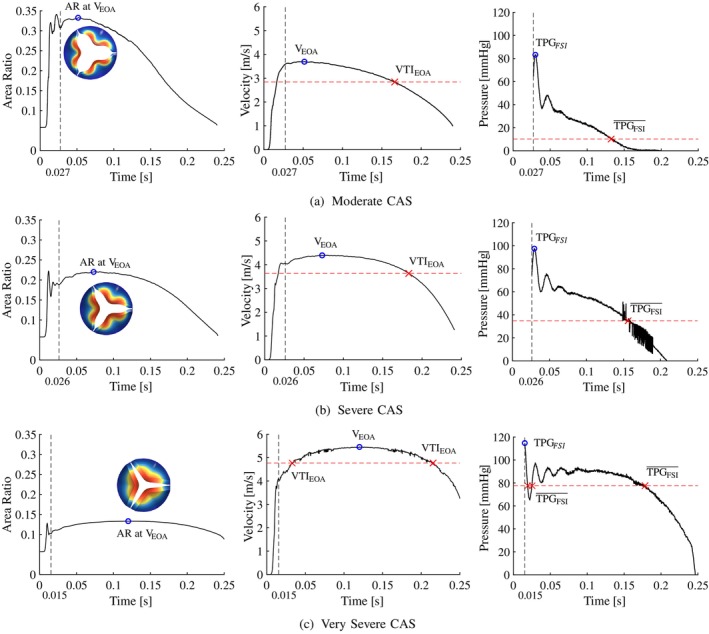
Calcific aortic stenosis. (a) Moderate, (b) severe and (c) very severe cases showing the area ratio (left), EOA velocity (middle) and TPG (right) profiles over the systolic period.

In Figure 4, the valve opening AR's (left), EOA velocity (middle) and pressure gradient (right) results for the moderate (Figure 4a), severe (Figure 4b) and very severe (Figure 4c) CAS cases are shown, and the results are summarised in Table 3. By analysing the AR profiles over the systolic period, in all cases, the AR increases rapidly during the opening phase of the valve, where it stabilises and is considered open at $t_{\mathrm{MC}}$ = 0.027 s, $t_{\mathrm{SC}}$ = 0.026 s and $t_{\mathrm{VSC}}$ = 0.015 s, respectively. The moderate CAS valve in Figure 4a has the longest opening phase with longer durations of AR oscillations compared to the more severe cases. However, peak systole is reached 0.021 s faster in the moderate case in Figure 4a compared to the severe case in Figure 4b, and 0.047 s faster in the severe case compared to the very severe case in Figure 4c due to the increased mobility of the leaflets at lower levels of severity. The AR and velocity profiles exhibit similar trends where the profile gradients decrease as the severity of CAS increases. This can be attributed to the reduced motion of the more severe leaflets, which enables the valve to reach peak systole later and remain at peak systole for longer periods before the valve starts to close. The corresponding peak systolic velocities at the EOA are $V_{\mathrm{EOA},\mathrm{MC}}=3.69$, $V_{\mathrm{EOA},\mathrm{SC}}=4.39$ and $V_{\mathrm{EOA},\mathrm{VSC}}=5.46\;m\cdot s^{-1}$, respectively. This indicates that the peak velocity increases with 0.7 and $1.07\;m\cdot s^{-1}$ as the CAS severity increases from moderate to severe and from severe to very severe, respectively. As peak systole is defined as the time when the EOA velocity reaches its maximum (i.e., $V_{\mathrm{EOA}}$), the pressure gradient at peak systole is denoted ${\mathrm{TPG}}_{\mathrm{PS}}$. From the results in Table 3, the maximum pressure gradient in the domain denoted ${\mathrm{TPG}}_{\mathrm{FSI}}$ is reached a minimum of 0.022 s before the model reaches peak systole in the moderate case, and a maximum of 0.105 s in the very severe case. Consequently, as the maximum pressure gradient is not recorded at peak systole, ${\mathrm{TPG}}_{\mathrm{PS}}$ is consistently lower than ${\mathrm{TPG}}_{\mathrm{FSI}}$ in all cases, with errors of 42.8, 37.3 and 24.3mm⋅Hg in the moderate, severe and very severe CAS cases, respectively. These findings indicate that relying solely on peak systolic conditions and $V_{\mathrm{EOA}}$ to determine the TPG using methods such as echo‐Doppler will result in underestimations in the current modelled examples.

**TABLE 3:** CAS simulation results for moderate, severe and very severe stenosis.

	Opening phase	Peak systole (i.e., $V=V_{\mathrm{EOA}}$)				True peak		Mean	
	*t* (s)	$t_{@V_{\mathrm{EOA}}}$ (s)	AR	$V_{\mathrm{EOA}}$ ($m\cdot s^{-1}$)	${\mathrm{TPG}}_{\mathrm{PS}}$ (mm⋅Hg)	$t_{@{\mathrm{TPG}}_{\mathrm{FSI}}}$ (s)	${\mathrm{TPG}}_{\mathrm{FSI}}$ (mm⋅Hg)	${\mathrm{VTI}}_{\mathrm{EOA}}$ ($m\cdot s^{-1}$)	$\bar{{\mathrm{TPG}}_{\mathrm{FSI}}}$ (mm⋅Hg)
MC	0.027	0.052	0.330	3.69	40.5	0.030	83.3	2.84	10.2
SC	0.026	0.073	0.219	4.39	60.2	0.029	97.5	3.63	34.9
VSC	0.015	0.120	0.134	5.46	90.5	0.015	114.8	4.77	77.6

The ${\mathrm{VTI}}_{\mathrm{EOA}}$ in the moderate, severe and very severe CAS cases are 2.84, 3.63 and $4.77\;m\cdot s^{-1}$, respectively. This indicates a relative percentage increase in ${\mathrm{VTI}}_{\mathrm{EOA}}$ values of 27.82% between moderate and severe cases, and 31.4% between severe and very severe cases. Similarly, for mean pressure gradients $\bar{{\mathrm{TPG}}_{\mathrm{FSI}}}$ estimated from the FSI results as 10.2, 34.9 and 77.6mm⋅Hg for the moderate, severe and very severe cases, respectively. The percentage increases in mean pressure gradients are 241% from moderate to severe cases and 122% from severe to very severe cases. From the pressure gradient profiles in Figure 4, the TPG profiles decrease proportionally to the valve area as the valve starts to close. As the moderate CAS valve opens and closes more rapidly compared to the more severe cases, the TPG profile is expected to have the steepest gradients with an almost linear decrease as the AR decreases. The severe and very severe CAS cases have similar TPG profile trends where the pressure gradient gradually decreases proportionally to the AR, and the TPG remains relatively constant during peak systole in the very severe case. As a result, the mean haemodynamic conditions are closer in magnitude to the peak haemodynamic conditions as the severity of CAS increases.

### 3.4 RAS

**FIGURE 5:**
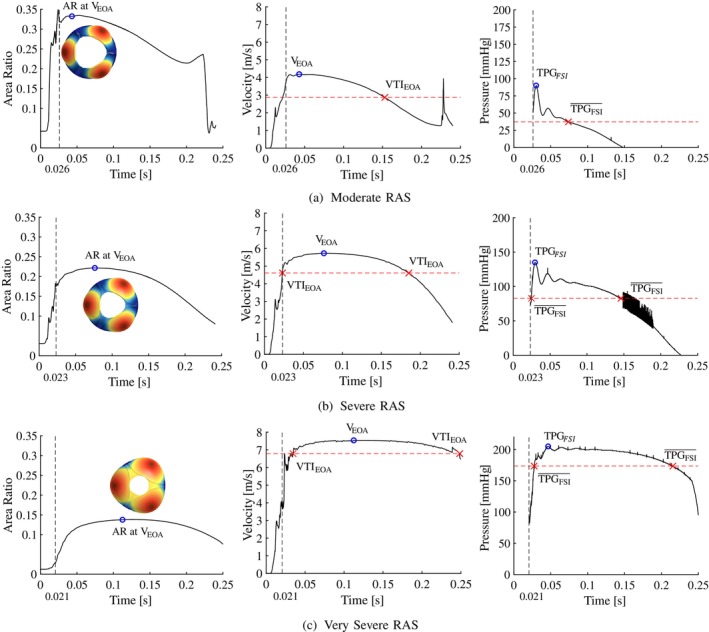
Rheumatic AS. (a) Moderate, (b) severe and (c) very severe cases showing the area ratio (left), EOA velocity (middle) and TPG (right) profiles over the systolic period.

Similar to the CAS cases, the valve opening ARs (left), EOA velocity (middle) and pressure gradient (right) results for the moderate (Figure 5a), severe (Figure 5b) and very severe (Figure 5c) RAS cases are shown in Figure 5 and the results summarised in Table 4. The AR profiles of the RAS cases exhibit similar trends to the CAS cases where the AR increases and fluctuates before it settles and is considered open at $t_{\mathrm{MR}}$ = 0.026 s, $t_{\mathrm{SR}}$ = 0.023 s and $t_{\mathrm{VSR}}$ = 0.021 s and, as the severity of RAS increases, the geometric opening area decreases, resulting in a decreased AR. However, from the AR profiles of the RAS cases in Figure 5 (left), it can be seen that these valve models reach fully open states later and exhibit fewer oscillations of the AR compared to the CAS cases. Peak systole is reached 0.033 s faster in the moderate RAS case in Figure 5a compared to the severe RAS case in Figure 5b, and 0.034 s faster in the severe RAS case compared to the very severe RAS case in Figure 5c. This shows that as the severity of RAS increases due the increase in commissural fusion length, more time is required for the valve to reach its maximum opening area, and the valves remain at peak systole for longer periods before the valve start to close. For peak systolic velocities ($V_{\mathrm{EOA}}$) in the respective moderate, severe and very severe RAS cases of 4.18, 5.73 and $7.54\;m\cdot s^{-1}$, $V_{\mathrm{EOA}}$ increase with 1.55 and $1.81\;m\cdot s^{-1}$ as the RAS severity increases from moderate to severe and from severe to very severe, respectively. The discrepancy between the time when the true peak TPG (i.e., ${\mathrm{TPG}}_{\mathrm{FSI}}$) is measured and the time of peak systole where ${\mathrm{TPG}}_{\mathrm{PS}}$ is measured ranges between 0.013 s in the moderate case and 0.063 s in the very severe case. Since ${\mathrm{TPG}}_{\mathrm{FSI}}$ precedes ${\mathrm{TPG}}_{\mathrm{PS}}$ in all cases, the peak systolic TPG is therefore 37, 30 and 7mm⋅Hg lower than the true peak TPG in the moderate, severe and very severe RAS cases, respectively. The TPG profile increases and decreases rapidly in the moderate case and more gradually in the severe case while in the very severe RAS case, the TPG remains relatively constant at 200mm⋅Hg before it reduces rapidly at the end of the systolic period.

**TABLE 4:** RAS simulation results for moderate, severe and very severe stenosis.

	Opening phase	Peak systole (i.e., $V=V_{\mathrm{EOA}}$)				True peak		Mean	
	*t* (s)	$t_{@V_{\mathrm{EOA}}}$ (s)	AR	$V_{\mathrm{EOA}}$ ($m\cdot s^{-1}$)	${\mathrm{TPG}}_{\mathrm{PS}}$ (mm⋅Hg)	$t_{@{\mathrm{TPG}}_{\mathrm{FSI}}}$ (s)	${\mathrm{TPG}}_{\mathrm{FSI}}$ (mm⋅Hg)	${\mathrm{VTI}}_{\mathrm{EOA}}$ ($m\cdot s^{-1}$)	$\bar{{\mathrm{TPG}}_{\mathrm{FSI}}}$ (mm⋅Hg)
MR	0.026	0.043	0.335	4.18	53.0	0.030	90.0	2.87	37.3
SR	0.023	0.076	0.222	5.73	105.0	0.029	135.0	4.61	82.8
VSR	0.021	0.110	0.138	7.54	200.3	0.047	207.0	6.79	173.6

The mean haemodynamic conditions for the respective RAS cases are calculated in terms of the ${\mathrm{VTI}}_{\mathrm{EOA}}$ as 2.87, 4.61 and $6.79\;m\cdot s^{-1}$ for the moderate, severe and very severe cases, respectively. This indicates a relative percentage increase in ${\mathrm{VTI}}_{\mathrm{EOA}}$ values of 60.6% between moderate and severe cases, and 47.3% between severe and very severe cases. The mean pressure gradients $\bar{{\mathrm{TPG}}_{\mathrm{FSI}}}$ estimated from the FSI results are 37.3, 82.8 and 173.6mm⋅Hg for the moderate, severe and very severe RAS cases, respectively. The mean TPG increases with 122% from moderate to severe cases and 109% from severe to very severe RAS cases. As observed in the CAS cases, the difference between the peak and mean velocity and pressure gradient magnitudes decreases with an increase in severity. This demonstrates that as severity increases, RAS cases exhibit greater relative increases in VTI changes between severity levels and smaller relative increases in pressure gradients compared to CAS cases.

### 3.5 General Comparison Between CAS and RAS

The effect of each disease morphology on the flow of blood through the valve is determined by comparing the results discussed in Sections 3.3 and 3.4. The generic valve models for the moderate, severe and very severe calcific and rheumatic cases at peak flow conditions are shown in Figures 4 and 5 and the ARs and AVAs summarised in Table 1. From Figures 4 and 5, it can be seen that for the same severity and similar AVAs and ARs, the two pathologies produce distinctly different opening morphologies where the calcific valve area has a more star‐like shape, and the rheumatic valves a more circular and triangular shape. Consequently, calcific valves have smaller hydraulic diameters than their equivalent RAS valves, resulting in lower Reynolds numbers and reduced frictional and secondary loss effects. This is evident in the previously mentioned results, where RAS valves exhibit substantially higher TPG values for the same ARs. Furthermore, it is evident that the rheumatic valve models experience more severe bulging due to the decreased stiffness of the leaflets, and reach fully open valve states faster and with smaller AR fluctuations during the opening phase compared to the calcific models.

The impeding effect of the diseased valves on the flow of blood is quantified by the mean flow rate (Equation 16) and the lost work (Equation 17) experienced by the blood flowing through the AV. The mean flow rate indicates the CO of the left ventricle‐AV combination, and the lost work indicates the additional work required by the ventricle to overcome the valvular pressure drop. These values (mean flow rate $\bar{Q}$ and lost work $W_{\text{lost}}$) are shown for the different morphologies and severities in Table 5. The peak and mean velocity magnitudes are used to determine the clinical TPG estimations according to Equation (15) and compared to the ground truth FSI results. These values are also shown in Table 5.

**TABLE 5:** Comparison between RAS and CAS simulation results for moderate, severe and very severe stenosis.

	Flow parameters		Peak				Mean		
	$\bar{Q}$ ($\mathrm{mL}\cdot s^{-1}$)	$W_{\text{lost}}$ (J)	$V_{\mathrm{EOA}}$ ($m\cdot s^{-1}$)	${\mathrm{TPG}}_{\mathrm{PS}}$ (mm⋅Hg)	${\mathrm{TPG}}_{\mathrm{FSI}}$ (mm⋅Hg)	${\mathrm{TPG}}_{\text{Clinical}}$ (mm⋅Hg)	${\mathrm{VTI}}_{\mathrm{EOA}}$ ($m\cdot s^{-1}$)	$\bar{{\mathrm{TPG}}_{\mathrm{FSI}}}$ (mm⋅Hg)	$\bar{{\mathrm{TPG}}_{\text{Clinical}}}$ (mm⋅Hg)
MC	298	0.34	3.69	40.5	83.3	54.5	2.84	10.2	32.3
SC	297	0.51	4.39	60.2	97.5	77.2	3.63	34.9	52.8
VSC	260	0.70	5.46	90.0	114.8	119.1	4.77	77.6	90.9
MR	300	0.36	4.18	53.0	90.0	70.0	2.87	37.3	33.0
SR	301	0.88	5.73	105.0	135.0	131.2	4.61	82.8	85.0
VSR	266	1.68	7.54	200.3	207.0	227.3	6.79	173.6	184.2

For a given severity of stenosis, identical inlet velocity waveforms were applied across different valve pathologies as the respective ventricular remodelling patterns and compensatory mechanisms associated with calcific versus rheumatic stenosis are not considered. Therefore, the SV describing the volume of blood pumped out of the LV at each heartbeat remains the same for both pathologies with the same stenosis severity. The moderate, severe and very severe cases have SV's of 71.1, 72.3 and $68.1\;\mathrm{mL}\cdot {\text{beat}}^{-1}$, respectively. For the same SV, the mean flow rate in Table 5 is higher in the RAS cases compared to the CAS cases. The lost work in the RAS cases are 5.6%, 42.0% and 58.3% higher compared to the CAS cases for moderate, severe and very severe cases, respectively, which alludes to the higher resistance the RAS valves offer to the flow of blood compared to the CAS valves. As a result, the LV must work harder in the presence of rheumatic stenosed valves to overcome the pressure drop caused by the pathology compared to the calcific stenosed valves of the same severity. Furthermore, the mean flow rate decreases with an increase in severity, which is proportional to the decrease in SV, and the lost work increases with an increase in severity due to the higher pressure gradients experienced by the more severe valves. The mean flow rate and lost work is, therefore, a function of the severity of stenosis (i.e., SV) and the type of stenosis described by the morphology of the valve.

The peak TPG recorded from the FSI data (${\mathrm{TPG}}_{\mathrm{FSI}}$) is compared to the TPG at the time of peak velocity (${\mathrm{TPG}}_{\mathrm{PS}}$) to determine the relative timing of the EOA velocity and pressure profiles as discussed in Sections 3.3 and 3.4. The peak and mean velocity and TPG measurements increase proportionally to the degree of stenosis in both cases. The moderate, severe and very severe peak EOA velocities ($V_{\mathrm{EOA}}$) in the RAS models are 0.49, 1.34 and $2.08\;m\cdot s^{-1}$ higher than the corresponding CAS models. Similarly, the mean EOA velocity (${\mathrm{VTI}}_{\mathrm{EOA}}$) in the RAS cases are 0.03, 0.98 and $2.02\;m\cdot s^{-1}$ higher than the CAS cases. Due to the high‐velocity profiles evident in the rheumatic cases, the peak and mean TPGs are expected to be higher than the calcific cases. The ${\mathrm{TPG}}_{\mathrm{FSI}}$ in the rheumatic cases are 7%, 28% and 45% higher than in the corresponding CAS case, while ${\mathrm{TPG}}_{\mathrm{FSI}}$ in the RAS cases are 73%, 58% and 55% higher than the respective CAS cases.

The pressure drop across an obstruction is governed by the energy loss coefficient and dynamic pressure of the flow field through the heart valve. In the context of AS, the different stenosis pathologies are associated with specific morphologies that produce distinct velocity profiles and pressure gradients due to varying hydraulic diameters which in turn dictate the flow passage Reynolds number that changes the flow passage energy loss coefficient. The FSI‐simulated pressure gradients can be compared to pressure gradients derived from the simplified Bernoulli equation using the velocities extracted from the same FSI simulations. By extracting velocities from the FSI simulations and applying the clinical TPG estimation method, discrepancies between the two approaches are identified that reveal inherent limitations of the simplified Bernoulli approach in the presence of different valve morphologies.

The clinical peak TPG (${\mathrm{TPG}}_{\text{Clinical}}$) and clinical mean TPG ($\bar{{\mathrm{TPG}}_{\text{Clinical}}}$) is calculated according to Equation (15) using $V_{\mathrm{EOA}}$ and ${\mathrm{VTI}}_{\mathrm{EOA}}$, respectively, and compared to the corresponding peak and mean FSI estimations as summarised in Table 5. In the calcific case, with respect to ${\mathrm{TPG}}_{\mathrm{FSI}}$, the ${\mathrm{TPG}}_{\text{Clinical}}$ underestimates the peak pressure gradient by 28.7mm⋅Hg in moderate and 20.3mm⋅Hg in severe and overestimates the peak pressure gradient by 4.3mm⋅Hg in the very severe case. Conversely, with respect to $\bar{{\mathrm{TPG}}_{\mathrm{FSI}}}$, the $\bar{{\mathrm{TPG}}_{\text{Clinical}}}$ are consistently overestimated by 22.2, 18 and 13mm⋅Hg in moderate, severe and very severe CAS cases, respectively. From these errors, it is clear that the clinical pressure estimation either under‐ or overestimates the peak and mean TPG depending on the severity of the calcifications.

In the rheumatic case, the ${\mathrm{TPG}}_{\text{Clinical}}$ is 20 and 3.8mm⋅Hg lower than ${\mathrm{TPG}}_{\mathrm{FSI}}$ for the moderate and severe cases and 20.3mm⋅Hg higher in the very severe case. The $\bar{{\mathrm{TPG}}_{\text{Clinical}}}$ is 4.3mm⋅Hg lower in the moderate case and 2.2 and 10.7mm⋅Hg higher in the severe and very severe cases when compared to $\bar{{\mathrm{TPG}}_{\mathrm{FSI}}}$. As with the calcific models, the clinical estimations in the RAS cases are inconsistent as it either underestimate or overestimate the peak and mean TPG depending on the severity of the disease. However, the clinical TPG estimation generally performs better in the RAS cases with smaller error magnitudes compared to the CAS cases.

In a clinical study by Handke et al. [45], the valve dynamics of 16 moderate to severe AS cases ($\mathrm{AVA}=\left(0.67\pm 0.24\right)\;{\mathrm{cm}}^{2}$) with normal LV function were analysed by TEE. The study reported an increase in time to reach the maximum opening state of the valve ranging between 80–180 ms and longer valve closure periods with estimated peak TPG of 40–130 mm·Hg. The TPG measured by invasive catheterisation was compared to non‐invasive Doppler TPG estimations for patients with AS with maximum systolic AVAs measured from CT images in a study by Franke et al. [18]. For AVAs of 0.31–1.77 cm^2^, the study reported peak‐peak TPG measurements of 5–160 mm·Hg and Doppler based TPG of 20–118 mm·Hg for peak systolic flow rates of $\left(296\pm 106\right)\;\mathrm{mL}\cdot s^{-1}$. In another study, Morany et al. [46] developed a 3D‐FSI model where the CAS valve model consists of calcium nodules segmented from patient‐specific CT images embedded in a generic valve model, and using the corresponding maximum Doppler based TPG as BCs in the domain. For the two respective calcified cases that were evaluated in that study with maximum AVA and TPG of (1) 0.82 cm^2^ and 74 mm·Hg and (2) 0.73 cm^2^ and 99 mm·Hg, peak EOA velocities of (1) 4.5 m·s^−1^ and (2) 5.5 m·s^−1^ were measured.

The CAS results simulated in the present work are in agreement with the clinical and numerical results obtained in the mentioned studies with peak simulated TPG (${\mathrm{TPG}}_{\mathrm{FSI}}$) of 83–115 mm·Hg, peak TPG at the time where peak EOA velocity is observed (${\mathrm{TPG}}_{\mathrm{PS}}$) of 41–90 mm·Hg, and peak clinical TPG estimations (${\mathrm{TPG}}_{\text{Clinical}}$) of 55–119 mm·Hg for AVAs ranging between 1.5 cm^2^ for moderate to 0.6 cm^2^ for very severe AS. The same FSI modelling methodology is followed in the RAS cases.

**FIGURE 6:**
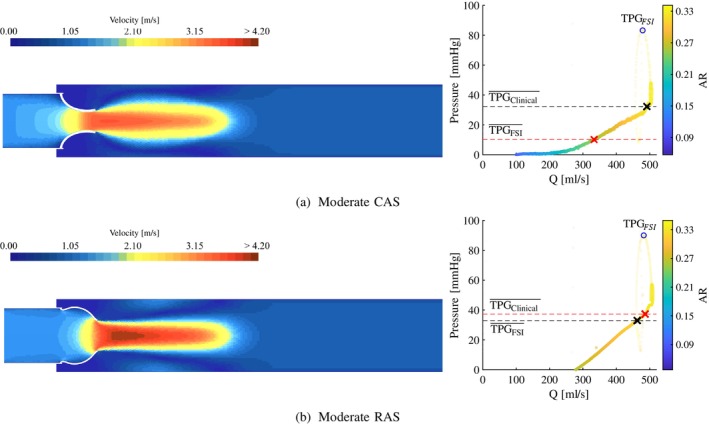
Moderate CAS and RAS peak systole velocity contour (left) and pressure gradients vs. volume flow (right). (a) Moderate calcific and (b) moderate rheumatic.

**FIGURE 7:**
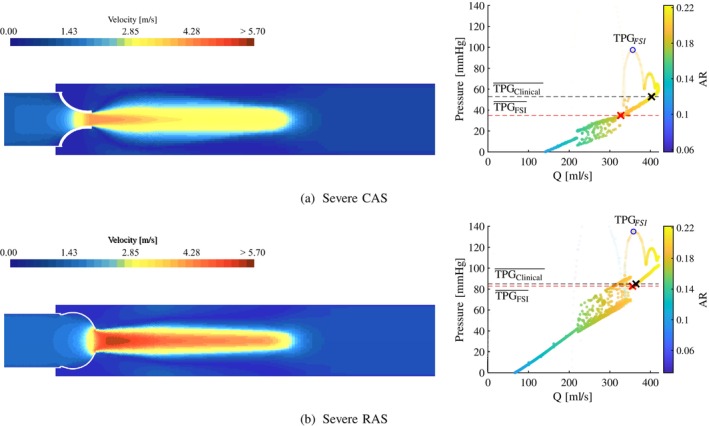
Severe CAS and RAS peak systole velocity contour (left) and pressure gradients vs. volume flow (right). (a) Moderate calcific and (b) moderate rheumatic.

**FIGURE 8:**
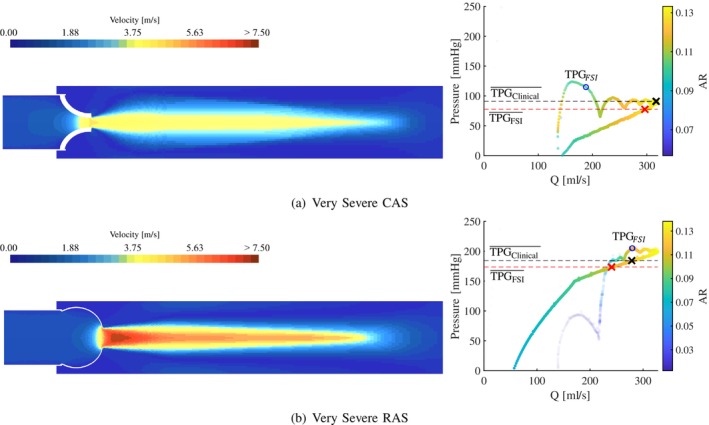
Very Severe CAS and RAS peak systole velocity contour (left) and pressure gradients vs. volume flow (right). (a) Moderate calcific and (b) moderate rheumatic.

In Figures 6, 7, 8, the velocity contours (left) and TPG‐Q relationships (right) are shown for each severity of AS along a plane orientated to cut through two of the valve leaflets. The velocity contour at peak systole is used to determine the velocity jet's shape and the EOA's location in the jet. The relationship between the pressure gradient and the flow rate is evaluated by presenting the TPG at each volume flow rate where the colour and the opacity of the data points are a function of the AR. The TPG‐Q relationships in these figures are generated by analysing the system curve for each data point as a function of AR. Therefore, each data point represents the instantaneous TPG and Q at a specific AR as the valve opens and closes and allows for the analysis of the combined *resultant system curve* (not to be confused with a typical fluid system quadratic system curve) for each case during the time when the flow is nearly or fully established.

As mentioned in Section 2.2, the stenosis present in the rheumatic valve models is only a function of the commissural fusion length, not the leaflet thickness. The stiffness of the leaflets in the RAS cases is therefore not influenced by the degree of stenosis to the same extent as in the CAS cases, and explains the excessive bulging of these valves and the elevated pressure gradients seen in Figure 5. Although the two pathologies are governed by the same severity when considering AR and SV, the increased jet velocity and pressure gradient profiles in the rheumatic cases can be attributed to the morphology of the valve area reduction. At peak systole, the morphology of the rheumatic valves closely resembles that of an orifice (although not perfectly circular), compared to a star‐like valve morphology of the calcific valves. The contraction mechanism drives the velocity jets in both the CAS and RAS cases and, therefore, friction at the valve walls proximal to the AVA, and sudden expansion distal to the AVA. As a result of the different valve morphologies, the velocity jet in the RAS cases is more concentrated and less dispersed compared to the CAS cases, as seen in the velocity contour plots at peak systole illustrated in Figures 6, 7, 8. The higher velocity jet core zones in the rheumatic valves result in larger magnitudes of shear stresses, higher energy dissipation, and, as a result, greater pressure gradients. From the velocity contour plots, it is evident that as the severity of the stenosis increases, the EOA moves proximal to the valve in the CAS cases in Figures 6a, 7a and 8a, and more distal in the RAS cases in Figures 6b, 7a and 8b.

From the TPG‐Q relationship in the moderately stenosed cases in Figure 6a,b (right), the calcific and rheumatic cases follow similar trends where the TPG increases with an increase in flow rate. At an AR of 0.33, the moderate CAS and RAS valves are fully open. For ARs between 0.28 and 0.33 and volume flow rates between 300 and 450 mL · s^−1^, the TPG increases at a rate of $0.14\;\mathrm{mm}\cdot \mathrm{Hg}\cdot s\cdot {\mathrm{mL}}^{-1}$ in the moderate CAS case in Figure 6a compared to $0.19\;\mathrm{mm}\cdot \mathrm{Hg}\cdot s\cdot {\mathrm{mL}}^{-1}$ in the moderate RAS case in Figure 6b. Therefore, for moderately stenosed valves the TPG increases at a 26% higher rate in the rheumatic case compared to the calcific case as it approaches peak systole.

As with the moderate valves, the TPG‐Q relationship in the severe cases in Figure 7a,b (right) also follow similar trends where the TPG in both disease types increases with an increase in flow rate. The severe models reach fully open states with an AR of approximately 0.22. For ARs between 0.17 and 0.22 and volume flow rates between 300 and 450 mL · s^−1^, the TPG‐Q gradient is $0.35\;\mathrm{mm}\cdot \mathrm{Hg}\cdot s\cdot {\mathrm{mL}}^{-1}$ in the severe CAS case in Figure 7a and $0.38\;\mathrm{mm}\cdot \mathrm{Hg}\cdot s\cdot {\mathrm{mL}}^{-1}$ in the severe RAS case in Figure 7b. This indicates that the TPG in the severe cases increases at an 8% higher rate in the rheumatic valve compared to the calcific valve as it approaches peak systole.

The velocity jet in the very severe calcific model in Figure 8a (left) develops downstream with a more dispersed jet compared to the very severe rheumatic case in Figure 8b (left). The TPG‐Q relationship in the very severe cases in Figure 8a,b (right) follow slightly different trends compared to the less severe cases. However, the TPG still increases as the volume flow rate increases. At least two trends with varying gradients can be identified for this severity. For flow rates below 150 mL·s^−1^, the very severe RAS model in Figure 8b has an approximate linear TPG‐Q gradient of $1.4\;\mathrm{mm}\cdot \mathrm{Hg}\cdot s\cdot {\mathrm{mL}}^{-1}$ and for volume flow rates between 200 and 350 mL·s^−1^ with ARs between 0.08 and 0.13, it has a gradient of $0.4\;\mathrm{mm}\cdot \mathrm{Hg}\cdot s\cdot {\mathrm{mL}}^{-1}$. Alternatively, the very severe CAS model in Figure 8a has gradients of $2.7\;\mathrm{mm}\cdot \mathrm{Hg}\cdot s\cdot {\mathrm{mL}}^{-1}$ for flow rates between 100 and 200 mL·s^−1^ and $0.58\;\mathrm{mm}\cdot \mathrm{Hg}\cdot s\cdot {\mathrm{mL}}^{-1}$ for volume flow rates between 200 and 350 mL·s^−1^ and ARs between 0.08 and 0.13. Therefore, for this severity the TPG increases at a rate that is 48% and 31% higher in the CAS case compared RAS case for volume flow rates of 100–200 and 200–350 mL·s^−1^, respectively. It is however important to note the difference in TPG magnitude between the two cases where the TPG in the very severe RAS model is approximately double the TPG in the very severe CAS model.

## 4 Limitations

The analyses presented in the current work are based on idealised representations of CAS and RAS with isolated pathological mechanisms (leaflet thickening due to calcification and commissural fusion). The AS cases are simplified models that may differ from what is seen in clinical settings, especially as the severity of the disease progresses where mixed lesions are commonly observed. Consequently, the magnitude of the pressure gradients, velocity profiles and energy losses estimated by the computational models in this study are expected to differ from the physiological conditions seen in clinical settings.

An isotropic Neo‐Hookean constitutive model for the leaflets is used based on its previous application in similar FSI studies [20, 26, 47] and its ability to reduce computational complexity while including sufficient non‐linearity [29]. The AV's deformation dynamics are governed by its material properties and constitutive model [48, 49], and directly influence the fluid dynamics in a two‐way coupled FSI model. Therefore, the use of an isotropic hyperelastic material model without experimental validation of the leaflet deformation patterns may limit the physiological accuracy of the predicted leaflet kinematics and the haemodynamic results presented in this numerical study.

The compliance of the aorta is not considered in this study, which can result in an overestimation of the pressure gradient when compared to physiological conditions. However, these effects are expected to be marginal when considering work by Pisani et al. [50] and Kivi et al. [51]. The focus of this study is to isolate the effects of the underlying pathologies on the haemodynamic environment of the AV, and not the secondary effects such as the compliance of the aorta.

The inlet BC is based on the flow rates approximated by a LPM that estimates the LV response to the varying degrees of stenotic valves. Although this approach is commonly used to approximate realistic BC's in FSI models using idealised geometries, it does not account for the different types of stenosis and the compensatory mechanisms of the LV in each case as the LPM and FSI models are not coupled. In clinical settings, different AS pathologies may result in specific cardiovascular adaptations, SV patterns and flow characteristics that can influence the valve's haemodynamic environment.

## 5 Conclusions

The results show that the haemodynamic behaviour of stenosed aortic heart valves depends on the disease's type and severity. CAS and RAS is modelled by considering the characteristics of the respective pathologies and, through the use of FSI modelling methodologies, the haemodynamic conditions in the AV lesions are determined by analysing the velocity profiles, pressure gradients and lost work in the domain. The results show that, for all severities of stenosis, the rheumatic valves offer higher resistance to the flow of blood compared to the calcific valves for similar flow rates and areas. The velocity profiles in the rheumatic cases have higher magnitudes with more concentrated and less dispersed velocity jets than the calcific cases. Consequently, the pressure gradients are higher in the rheumatic cases than in the calcific cases. Velocity reaches a maximum at the *vena contracta* (i.e., EOA) located proximal to the valve in the CAS cases and distal to the valve in the RAS cases. The clinical estimation of the pressure gradient is considered to determine the impact that the type of stenosis has on the estimated TPG according to the simplified Bernoulli equation used in clinical practice. The results emphasise the insensitivity of the clinical estimation on valve morphology as it either under‐ or overestimates the TPG depending on the type and the severity of the valve lesion. Therefore, it can be concluded from this numerical study, considering the dynamics of the opening and closing of the AV in isolation, that the morphology of the valve has an effect on the haemodynamic conditions of stenosed AVs and that the clinical estimation generally performs better in predicting the TPG in the RAS cases compared to the CAS cases.

In addition, due to the lack of information on RAS and how it compares to CAS, the results from this study provide a theoretical baseline for the haemodynamic behaviour of stenosed AVs with different pathologies and severities, which can be used to inform further computational and clinical studies. In particular, due to the common misrepresentation of AS as a single pathology (i.e., CAS), this study provides invaluable insight into the potential implications of disregarding the different pathologies that cause AS during analyses and, as a result, the limitations of the simplified Bernoulli equation used in current clinical practice from a purely numerical perspective.

## Author Contributions

Model development: Lindi Grobler Kock and Ryno Laubscher. Data analysis: Lindi Grobler Kock, Ryno Laubscher, Johan van der Merwe, Martin P. Venter and Philip G. Herbst. Original draft preparation: Lindi Grobler Kock. Review and editing: Lindi Grobler Kock, Ryno Laubscher, Martin P. Venter, Johan van der Merwe, Anton F. Doubell and Philip G. Herbst. Visualisation: Lindi Grobler Kock, Ryno Laubscher and Johan van der Merwe. Project administration: Lindi Grobler Kock, Ryno Laubscher, Johan van der Merwe and Philip G. Herbst. Funding acquisition: Lindi Grobler Kock and Ryno Laubscher. All authors contributed to the critical review and revision of the manuscript.

## Funding

This work was supported by the National Research Foundation of South Africa (PMDS22071138485).

## Ethics Statement

The authors have nothing to report.

## Conflicts of Interest

The authors declare no conflicts of interest.

## Data Availability

The data that support the findings of this study are available from the corresponding author upon reasonable request.
